# SREBP2 promotes the viability, proliferation, and migration and inhibits apoptosis in TGF-β1-induced airway smooth muscle cells by regulating TLR2/NF-κB/NFATc1/ABCA1 regulatory network

**DOI:** 10.1080/21655979.2022.2026550

**Published:** 2022-01-17

**Authors:** Yuebin Wang, Huike Yang, Xian Su, Anqiang Cao, Feng Chen, Peng Chen, Fangtao Yan, Huirong Hu

**Affiliations:** a Department of Respiratory and Critical Care Medicine, Chengdu Third People’s Hospital, Chengdu, China; b Department of Anatomy, Harbin Medical University, Harbin, China; c Department of Cardiothoracic Surgery, Meishan People’s Hospital, Meishan, China; d Department of Cardiothoracic Surgery, Chengdu Third People’s Hospital, Chengdu, China

**Keywords:** SREBP2, TGF-Β1, TLR2, NFATC1, ABCA1

## Abstract

Asthma is a respiratory disease with complex pathogenesis. Sterol-responsive element-binding proteins 2 (SREBP2) was found to bind to promoter sequences of ABCA1 to suppress ABCA1 promoter activity. This study aimed to explore the expression level of SREBP2 and ATP-binding cassette transporter A1 (ABCA1), and their effects on the development of airway smooth muscle cells (ASMCs) in asthma. ASMCs were treated with different concentrations of TGF-β1 (0, 0.5, 1, 5 and 10 ng/mL). Short hairpin SREBP2 (shSREBP2), SREBP2, shABCA1 or ABCA1 were transfected into ASMCs. Cell viability, proliferation, apoptosis, migration, and the expression of SREBP2, ABCA1 and related pathway proteins were detected by MTT assay, Brdu staining, flow cytometer, Transwell assay, qRT-PCR, and Western blotting, respectively. The results showed that TGF-β1 increased the viability, proliferation, migration and inhibited apoptosis in ASMCs. Moreover, TGF-β1 also decreased the expression of ABCA1, cleaved caspase-3, cleaved PARP, E-cadherin, and increased the expression of vimentin, TLR2, p-p65 and NFATc1. SREBP2 knockdown alleviated these TGF-β1-induced changes. SREBP2 overexpression inhibited ABCA1 expression and apoptosis, and promoted cell migration and the expression of TLR2, p-p65, NFATc1 in ASMCs. ABCA1 overexpression alleviated these SREBP2-induced promoting and inhibition effects. In conclusion, SREBP2 activates TLR2/NF-κB/NFATc1 regulatory network and promotes TGF-β1-induced cell movement through inhibiting ABCA1 expression.

## GRAPHIC ABSTRACT

**Figure:**
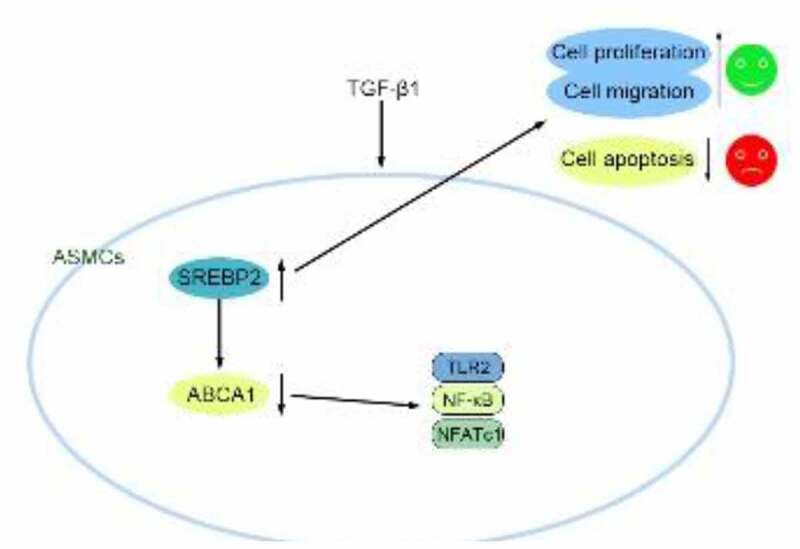

## Introduction

Asthma is respiratory disease with an increasing incidence year by year [1]. In the early stage of asthma, most patients experience allergic inflammation. In the later stage of asthma, the patients’ airways may be obstructed due to smooth muscle hyperplasia, leading to airway stenosis, airway hyperresponsiveness and airway remodeling, and ultimately chest tightness and wheezing, and even death [2,3]. Therefore, it is essential to study the pathogenesis of asthma and to explore new treatments to reduce the sufferings of patients.

One of the important mechanisms of asthma is airway remodeling caused by abnormal proliferation of airway smooth muscle [4]. In airway remodeling, airway wall thickening leads to airway stenosis and increased airway resistance [5]. When the airway wall thickens, airway smooth muscle cells (ASMCs) proliferate [6]. Transforming growth factor (TGF)-β1 is involved in the fibrosis of severe asthma by promoting fibroblast proliferation and synthesis of fibronectin. Moreover, the mRNA level of TGF-β1 expressed in the airway mucosal layer of asthmatic patients was significantly decreased, indicating that TGF-β1 could induce the occurrence of asthma. However, *in vitro* experiment has shown that TGF-β1 also stimulates the proliferation and migration of ASMCs [7]. Therefore, studying ASMCs *in vitro* is an important way to study the pathogenesis of asthma, and TGF-β1 can be used to establish *in* *vitro* asthma cell models.

As a membrane-bound nuclear transcription factor, sterol-responsive element-binding proteins 2 (SREBP2) regulates low-density lipoprotein (LDL) receptor expression by detecting intracellular cholesterol levels to maintain cellular cholesterol uptake and synthesis in a balanced state [8,9]. SREBP2 forms a complex with nucleotide oligomerization domain-like receptor protein 3 (NLRP3) and promotes NLRP3 inflammasome activation, thereby increasing inflammation *in vitro* and *in vivo* [10]. Inhibition of SREBP2 expression can prevent lung injury caused by viral infection [11]. In addition, SREBP2 actively participates in lipid metabolism. Interestingly, asthma is also associated with lipid metabolism, such as phospholipid and eicosanoids. Almstrand AC et al. found that patients with smoking-induced asthma exhale significantly lower unsaturated or saturated phospholipid species in respiratory particles than healthy patients who do not smoke. Moreover, asthma leads to abnormal arachidonic acid metabolic pathways [12]. Therefore, we speculated that SREBP2 may be associated with asthma, but the specific mechanism of SREBP2 in asthma is unknown.

ATP-binding cassette transporter A1 (ABCA1) is a key gene that controls the intracellular cholesterol, phospholipid efflux and reverse cholesterol transport [13]. ABCA1 promotes the efflux of free intracellular cholesterol and phospholipids in an ATP-dependent manner and binds to apolipoprotein to form high-density lipoprotein (HDL) [14]. Sterol loss in vascular endothelial cells has been reported to activate SREBP2 and decrease ABCA1 mRNA levels, and SREBP2 binds to ABCA1 promoter sequences and inhibits ABCA1 promoter activity [15]. However, whether SREBP2 also can modulate the activity of ABCA1 in asthma is unknown.

In this study, we speculate that SREBP2 is a crucial regulator for asthma and may be involved in asthma by regulating cell motility and the activity of ABCA1. Therefore, the present study used TGF-β1 stimulation to prepare ASMCs cell models *in vitro* to investigate the specific role of SREBP2 in the mechanism of airway remodeling in asthma and the regulation of ABCA1.

## Materials and methods

### Cell treatment

Human ASMCs from tracheal tissues were purchased from Procell (Wuhan, China) and cultured in Dulbecco’s Modified Eagle Medium (DMEM, Regal, Shanghai, China) with 10% fetal bovine serum (FBS, Thermo Fisher Scientific, Waltham, USA) and penicillin (Procell) at 37°C to a cell density of 70–80% [16]. Then, ASMCs were re-cultured in serum-free DMEM for another 18 h for cell starvation to achieve cell synchronization.

Starved ASMCs were treated with different concentrations of TGF-β1 (0, 0.5, 1, 5 and 10 ng/mL, Sigma-Aldrich, St. Louis, USA) for 48 h to establish asthma cell models *in vitro*.

## Cell transfection

shSREBP2, shABCA1, SREBP2 vector, ABCA1 vector or their empty vector were synthesized by Sangon Biotech Co., Ltd (Shanghai, China) and transfected into ASMCs or 5 ng/ml TGF-β1-stimulated ASMCs by Lipofectamine 3000 (Thermo Fisher Scientific). Moreover, ASMCs without any treatment served as the control group.

## Quantitative real-time polymerase chain reaction (qRT-PCR)

The total RNA was extracted from ASMCs by using TRIzol^TM^ Reagent (Thermo Fisher Scientific) and reverse transcribed into cDNA by mRNA Selective PCR Kit (TaKaRa, Dalian, China). PCR amplification was performed with PCR MasterMix (Solarbio, Beijing, China) to assess SREBP2 and ABCA1 expression level. The conditions of PCR amplification were: pre-denatured at 95°C for 5 min; denatured at 95°C for 30 s, annealed at 55°C for 20 s, extended at 72°C for 20 s, repeated 40 times; extended at 72°C for 5 min. The sequences of SREBP2 were: sense primer, 5ʹ-TCCGCCTGATCCGATGTAC-3ʹ; anti-sense primer: 5ʹ-TGCACAATCAGCCAGGTTCA-3ʹ. The sequences of ABCA1 were: sense primer, TCCTCCTGGTGAGTGCTTTG-3ʹ; anti-sense primer: 5ʹ-GGGACTCCTCTCAAAAGGGC-3ʹ. The glyceraldehyde-3-phosphate dehydrogenase (GAPDH) was as an internal reference, and its sequences were: sense primer, 5ʹ- TGAACGGGAAGCTCACTGG −3ʹ, anti-sense primer: 5ʹ-TCCACCACCCTGTTGCTGTA −3ʹ. The expression level in this study was calculated by the 2^−ΔΔCt^ method [17].

## Western blotting

The total protein was extracted from cells by protein lysis buffer (TaKaRa, Dalian, China) and quantified by Bradford method. An equal amount of protein (20 μg/lane) was separated by SDS-PAGE and then transferred onto polyvinylidene fluoride (PVDF) membranes by electrophoresis. After blocking, proteins were incubated with primary antibodies and horseradish peroxidase (HP)-labeled secondary antibody (ab191866, 1:2000, Abcam, Cambridge, USA), rinsed in the blocking solution, developed in color development reagent, and imaged in gel imaging system. The primary antibodies were SREBP2 (ab30682, 1:1000, 126 kDa), ABCA1 (ab125064, 1:1000, 254 kDa), GAPDH (ab181602, 1:1000, 36 kDa), cleaved caspase-3 (ab2302, 1:1000, 17 kDa), cleaved poly ADP-ribose polymerase (PARP, ab32064, 1:1000, 27 kDa), E-cadherin (E-cad, ab40772, 1:1000, 97 kDa), vimentin (ab8978, 1:1000, 57 kDa), toll-like receptor (TLR2, ab213676, 1:1500, 89 kDa), p-p65 (ab86299, 1:1000, 60 kDa), p65 (ab19870, 1:1500, 72 kDa), and nuclear factor-activated T cell 1 (NFATc1, ab2796, 1:1500, 101 kDa). The images were analyzed using Quantity One software.

## Methylthiazolyldiphenyl-tetrazolium bromid (MTT) assay

Cells were seeded on 96-well cell culture plate (at a density of 3–5 × 10^4^ cells/mL) to incubate for 48 h. Cell suspension was added with MTT solution (10 mg/mL, Beyotime, Shanghai, China) to incubate for 4 h, and then added with dimethyl sulfoxide (DMSO) to shock for 10 min. The absorbance (OD 490 nm) was measured by the spectrophotometer (Laspec, China).

## Bromodeoxyuridine (Brdu) staining

Cells were cultured for 48 h and added with BrdU solution (30 μg/L, Beyotime) and incubated for 40 min at 37°C. Then, cells were fixed and incubated with anti-BrdU antibody (1:50, Trevigen, USA). After washing, cells were stained in hematoxylin (Beyotime) for 30 min in the dark at room temperature. The BrdU positive cells were randomly counted to calculate the labeling index under the microscope (Leica, Germany) [16].

## Transwell assay

Cells were incubated for 48 h and added into the upper chamber of Transwell. The lower chamber of Transwell was added with culture medium. Cells were cultured for 24 h in Transwell, fixed with formaldehyde and stained with crystal violet (Beyotime). Scattered cells were removed with a cotton swab. The number of transmembrane cells was observed and counted under a microscope (Leica).

## Apoptosis

Cultured cells were stained by Hoechst Staining Kit (Beyotime), digested with 0.25% trypsin (Beyotime), and then added with Annexin-V and Propidium solution (Beyotime) and incubated for 20 min. The apoptosis ratio was detected by a flow cytometry (Beckman Coulter, USA). The results were expressed as the sum of early and late apoptotic rate.

## Statistical analysis

The results of experiments were analyzed using the SPSS 21.0 software (SPSS Inc., USA) and displayed with mean ± standard deviation (SD). Analysis of variance (ANOVA) was used with Bonferroni test in this study. If *p* < 0.05, the results were statistically significant.

## Results

The specific mechanism of action of SREBP2 in asthma with complex pathogenesis remains unclear. The effect of SREBP2 expression and its target factor ABCA1 on ASMCs was explored. *In vitro* asthma cell model was established by ASMCs treated with different concentrations of TGF-β1. Then, cell viability, proliferation, apoptosis, and migration, as well as the expression of SREBP2, ABCA1 and pathway proteins were analyzed after cell model transfected with shSREBP2, shABCA1 or ABCA1. The results indicated that SREBP2 induces the activation of TLR2/NF-κB/NFATc1 regulatory network and promotes TGF-β1-induced the changes of cell motility by inhibiting ABCA1 expression.

## TGF-β1 increased SREBP2 level and decreased ABCA1 level

**Figure 1.**
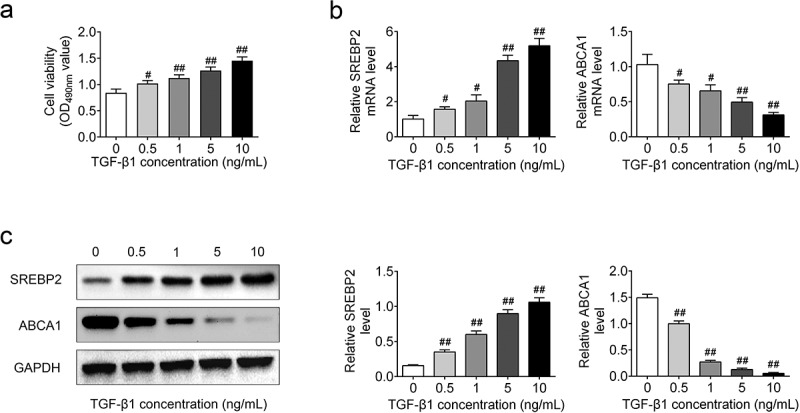
TGF-β1 increased SREBP2 level and decreased ABCA1 level in ASMCs. Different concentrations of TGF-β1 (0, 0.5, 1, 5 and 10 ng/mL) were used to treat ASMCs. (a) Cell viability, the relative SREBP2 and ABCA1 (b) mRNA level and (c) expression level was detected by MTT assay, qRT-PCR, and Western blotting. ^#^ *p* < 0.05, ^##^ *p* < 0.01; ^#^ compare with the 0 ng/mL TGF-β1 group.

In this study, ASMCs were treated with different concentration of TGF-β1 to establish an *in vitro* cell model of asthma. As shown in Figure 1(a), cell viability was gradually enhanced with the increase of TGF-β1 concentration (*p* < 0.05). In addition, SREBP2 expression levels were increased in a dose-dependent manner in TGF-β1-stimulated ASMCs (*p* < 0.05, Figure 1(b,c)). In contrast, TGF-β1 gradually decreased the expression level of ABCA1 with the increase of TGF-β1 concentration (*p* < 0.05, Figure 1(b,c)). Altogether, these results indicated that TGF-β1 induces the increases in SREBP2 expression and cell viability as well as the reduction in ABCA1 levels in ASMCs.

## SREBP2 knockdown inhibited proliferation, migration and promoted apoptosis

**Figure 2.**
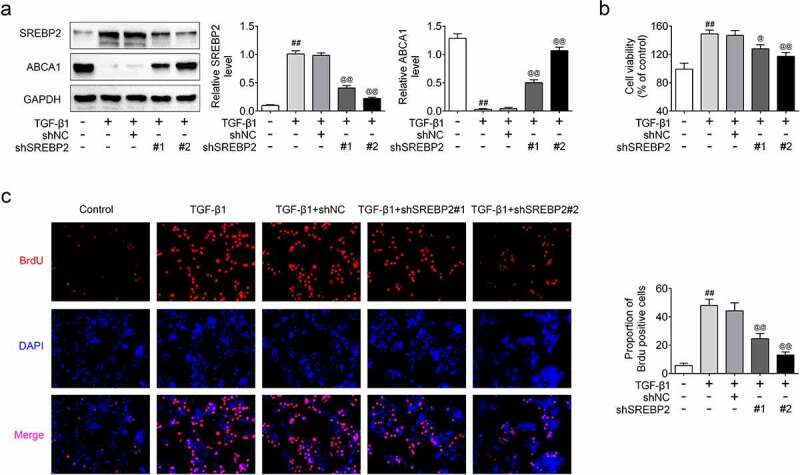
SREBP2 knockdown inhibited cell proliferation in ASMCs. shSREBP2 or shNC were transfected into ASMCs, or 5 ng/mL TGF-β1 were treated with ASMCs. (a) The relative SREBP2 and ABCA1 level, (b) cell viability, and (c) cell proliferation was detected by Western blotting, MTT assay and Brdu staining. ^##^ *p* < 0.01; ^#^ compare with the control group. ^@^ *p* < 0.05, ^@@^ *p* < 0.01; ^@^ compare with the TGF-β1 + shNC group.

**Figure 3.**
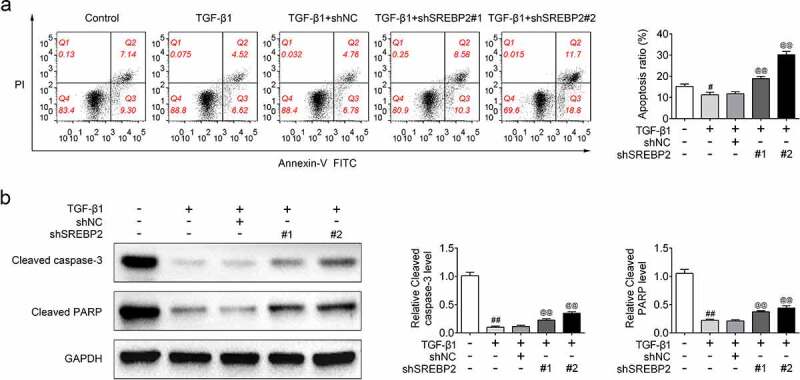
SREBP2 knockdown promoted apoptosis in ASMCs. shSREBP2 or shNC were transfected into ASMCs, or 5 ng/mL TGF-β1 was treated with ASMCs. (a) Apoptosis and (b) apoptosis-related proteins, caspase-3 and cleaved PARP levels were detected by flow cytometer and Western blotting. ^#^ *p* < 0.05, ^##^ *p* < 0.01; ^#^ compare with the control group. ^@^ *p* < 0.05, ^@@^ *p* < 0.01; ^@^ compare with the TGF-β1 + shNC group.

**Figure 4.**
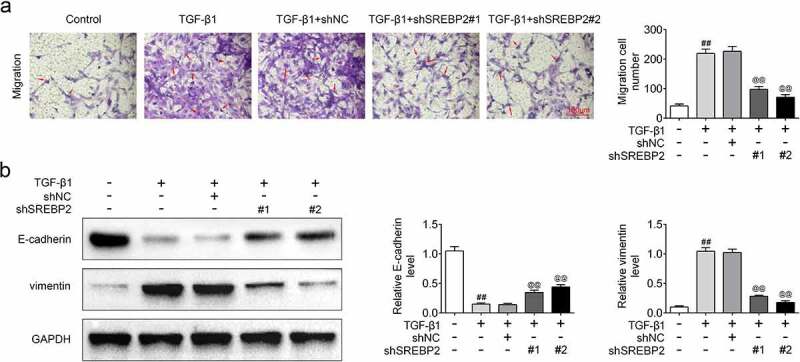
SREBP2 knockdown inhibited cell migration in ASMCs. shSREBP2 or shNC was transfected into ASMCs, or 5 ng/mL TGF-β1 was treated with ASMCs. (a) Cell migration and (b) migration-related proteins, E-cad and vimentin levels were detected by Transwell assay and Western blotting. ^#^ *p* < 0.05, ^##^ *p* < 0.01; ^#^ compare with the control group. ^@^ *p* < 0.05, ^@@^ *p* < 0.01; ^@^ compare with the TGF-β1 + shNC group.

In order to examine the regulatory effect of SREBP2 on asthma cell model, shSREBP2 or shNC were transfected into ASMCs. The transfection efficiency of shSREBP2 was detected by qRT-PCR and as shown in Figure 2(a), SREBP2 expression was down-regulated in shSREBP2 group (*p* < 0.05). Meanwhile, the ABCA1 expression level was increased in the TGF-β1 + shSREBP2 group compared with TGF-β1 + shNC group (*p* < 0.05, Figure 2(a)), demonstrating that SREBP2 targeted the expression of ABCA1. Moreover, Figure 2(b) shows that shSREBP2 alleviated the TGF-β1-induced increase in cell viability (*p* < 0.05). Similarly, Brdu staining result also showed that shSREBP2 inhibited TGF-β1 stimulated cell proliferation (*p* < 0.05, Figure 2(c)). The apoptotic rate of ASMCs was decreased after TGF-β1 treatment, while these decreases were reversed by SREBP2 knockdown (*p* < 0.05, Figure 3(a)). Compared with control group, cleaved caspase-3 and cleaved PARP expression levels were significantly reduced in TGF-β1 group. Then, the reduction of cleaved caspase-3 and cleaved PARP expression were alleviated in shSREBP2 transfected ASMCs (*p* < 0.05, Figure 3(b)). In addition, Figure 4(a) shows that TGF-β1 increased the number of migrating cells, whereas shSREBP2 inhibited these increases in ASMCs (*p* < 0.05). The Western blotting results in Figure 4(b) also shows that E-cad and vimentin (the proteins related to cell migration) expression was affected by TGF-β1 and shSREBP2. Specifically, TGF-β1 increased vimentin level and decreased E-cad level, whereas shSREBP2 markedly alleviated these changes (*p* < 0.05, Figure 4(b)).

**Figure 5.**
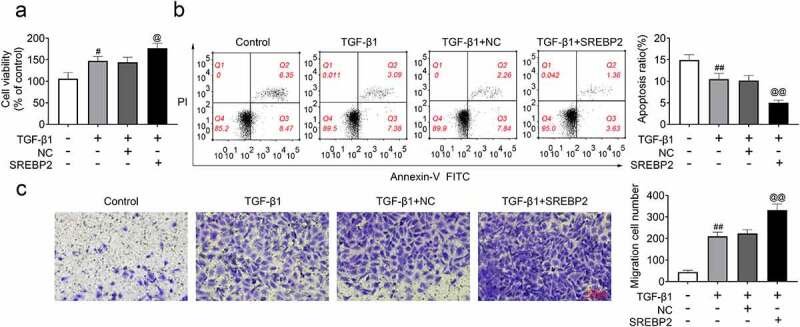
SREBP2 promoted cell viability, migration, and inhibited apoptosis in ASMCs. SREBP2 vector or empty vector were transfected into ASMCs, or 5 ng/mL TGF-β1 was treated with ASMCs. (a) Cell viability, (b) apoptosis and (c) cell migration were detected by MTT assay, flow cytometer and Transwell assay. ^#^ *p* < 0.05, ^##^ *p* < 0.01; ^#^ compare with the control group. ^@^ *p* < 0.05, ^@@^ *p* < 0.01; ^@^ compare with the TGF-β1 + shNC group.

In contrast, TGF-β1-induced increases in cell viability and migration were continuously enhanced when SREBP2 was overexpressed in ASMCs (*p* < 0.01, Figure 5(a,c)). Meanwhile, TGF-β1-induced decreases in apoptosis were alleviated by the transfection of SREBP2 in ASMCs (*p* < 0.01, Figure 5(b)). Therefore, the above results suggested that SREBP2 knockdown attenuates TGF-β1-induced the increases in cell proliferation and migration, as well as the reduction in apoptosis in ASMCs.

## SREBP2 activated the TLR2/NF-κB/NFATc1 pathway through inhibiting ABCA1 expression

**Figure 6.**
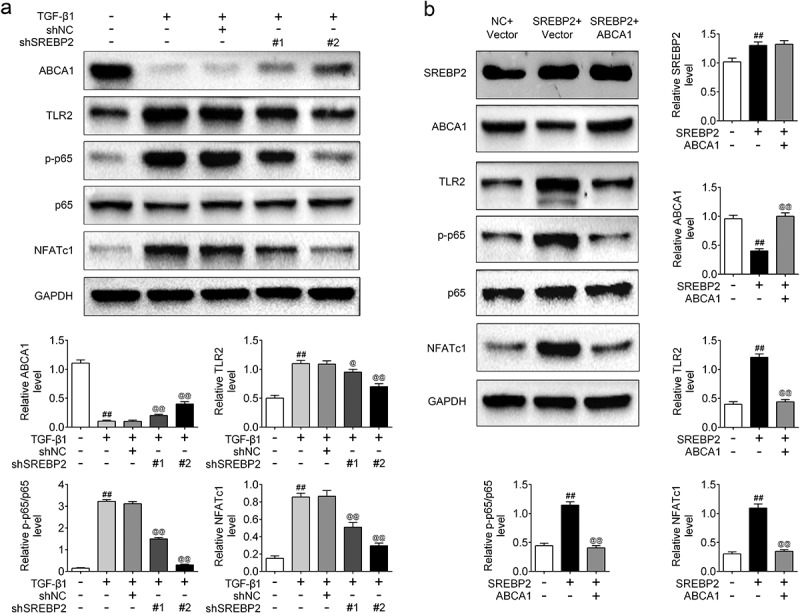
SREBP2 activated the TLR2/NF-κB/NFATc1 pathway by inhibiting the expression of ABCA1. shSREBP2, SREBP2 vector or ABCA1 vector were transfected into ASMCs, or 5 ng/mL TGF-β1 was treated with ASMCs. (a) The level of ABCA1, TLR2, p-p65, p65 and NFATc1, and (b) SREBP2, ABCA1, TLR2, p-p65, p65 and NFATc1 were detected by Western blotting. ^##^ *p* < 0.01; ^#^ compare with the control group. ^@^ *p* < 0.05, ^@@^ *p* < 0.01; ^@^ compare with the TGF-β1 + shNC or SREBP2 group.

In order to investigate the specific mechanism by which SREBP2 regulates cell motility, Western blotting was used to analyze the expression of several pathway-related proteins that may be involved in the development of asthma. As shown in Figure 6(a), TGF-β1 significantly down-regulated ABCA1 expression, and shSREBP2 up-regulated ABCA1 expression (*p* < 0.05). In addition, TGF-β1 significantly up-regulated the expression of TLR2, p-p65 and NFATc1. However, the transfection of shSREBP2 in TGF-β1-treated ASMCs resulted in the decreases in TLR2, p-p65 and NFATc1 expression (*p* < 0.05). Then, SREBP2 vector and ABCA1 vector were transfected into ASMCs to verify the regulatory relationship and mechanism of SREBP2 and ABCA1. Figure 6(b) shows that SREBP2 decreased ABCA1 expression levels. After simultaneous transfection of SREBP2 and ABCA1, the reduction of ABCA1 was restored, but the expression of SREBP2 did not change, indicating that the addition of ABCA1 cannot change the expression of SREBP2, but SREBP2 can change the expression of ABCA1. Thus, the target gene of SREBP2 is ABCA1. Moreover, TLR2, p-p65 and NFATc1 levels were increased by SREBP2 overexpression, whereas these increases were inhibited when ABCA1 was overexpressed in ASMCs (*p* < 0.05). Therefore, SREBP2 modulates ABCA1 expression to activate TLR2/NF-κB/NFATc1 pathway.

## SREBP2 promoted TGF-β1-induced cell motility through regulating ABCA1 expression

**Figure 7.**
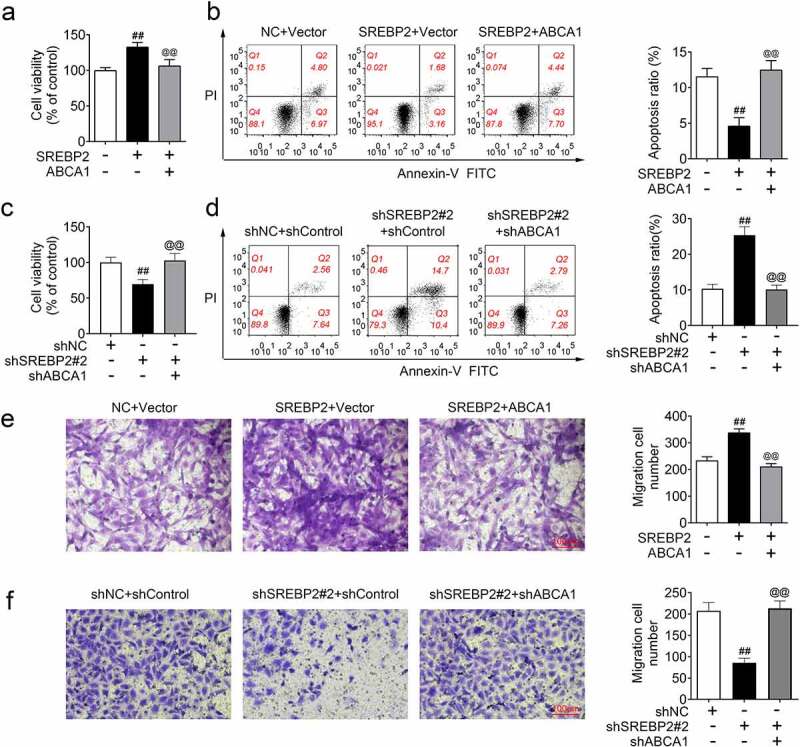
SREBP2 promoted TGF-β1-induced cell motility through regulating ABCA1 expression. shSREBP2, SREBP2 vector, shABCA1 or ABCA1 vector were transfected into ASMCs. (a and c) Cell viability, (b and d) apoptosis and (e and f) cell migration was detected by MTT assay, flow cytometer and Transwell assay. ^##^ *p* < 0.01; ^#^ compare with the control group. ^@@^ *p* < 0.01; ^@^ compare with the SREBP2 group or the shSREBP2 group.

In order to continue to investigate the specific mechanism by which SREBP2 regulated cell motility, SREBP2 vector, ABCA1 vector, shSREBP2 or shABCA1 were transfected into ASMCs. As shown in Figure 7(a), SREBP2 overexpression increased cell viability, and ABCA1 overexpression alleviated these increases in cell viability (*p* < 0.05). As shown in Figure 7(b), the apoptosis rate was decreased in the SREBP2 group compared with the control group, and increased in the SREBP2 + ABCA1 group compared with the SREBP2 group (*p* < 0.05). In contrast, ABCA1 knockdown inhibited shSREBP2-induced decreases in cell viability and increases in apoptosis (*p* < 0.05, Figure 7(c,d)). Figure 7(e) shows that the number of migrated cells in the SREBP2 group was higher than that in the control group, and also higher than that in the SREBP2 + ABCA1 group (*p* < 0.05). As shown in Figure 7(f), SREBP2 knockdown reduced the number of migrated cells, whereas shABCA1 inhibited these reductions (*p* < 0.05). Hence, SREBP2 regulates cell motility by targeting ABCA1 expression.

## Discussion

The greatest feature of asthma is the development of airway inflammation and airway remodeling [3,18]. The proliferation and migration of ASMCs is an important pathological base for airway remodeling in asthma [19]. TGF-β1 is able to stimulate the proliferation and migration of ASMCs [7]. Notability, the TGF-β1/Smad signaling pathway is a classic pathway regulating the involvement of ASMC in the pathogenesis of asthma [20]. Chen et al. reported that inhibition of the TGF-β1/Smad signaling pathway effectively reduced the level of airway remodeling in asthma [21]. Therefore, the above studies are sufficient to show that TGF-β1 causes asthma. In this study, we established an *in vitro* asthmatic cell model by stimulation with different concentrations of TGF-β1 and found that TGF-β1 could dose-dependently increase the viability, proliferation, and migration of ASMCs.

During airway remodeling, growth factors and inflammatory transmitters further exacerbate the asthmatic condition by stimulating ASMCs to cause airway hypertrophy, contraction, and release of inflammation factor [18,22]. Qiang et al. reported that inflammation significantly affects the expression of SREBP2 and alters the homeostasis of intracellular cholesterol [23]. Furthermore, the expression of LDL receptor in cells is regulated by SREBPs. As an inflammatory factor, TGF-β1 increases the activity of LDL receptor promoter and promotes the expression of LDL receptor. The inflammatory factors can interfere with the negative feedback regulation of LDL receptors by regulating SREBP2, leading to the formation of foam cells [24]. ASMCs are one of the important sources of foam cells [8,9]. Therefore, we hypothesized that SREBP2 is regulated by TGF-β1 and involved in ASMC motility and even in the phenotypic transformation of ASMCs. In the present study, the results confirmed that SREBP2 expression could be up-regulated by TGF-β1, and SREBP2 enhanced TGF-β1-induced cell motility, suggesting that SREBP2 may be a proinflammatory factor for asthmatic ASMCs.

ABCA1 is a determinant of plasma HDL levels and macrophage cholesterol levels. ABCA1 dysfunction in human results in reduced plasma HDL levels and converts cholesterol deposited in macrophages into foam cells [25]. The elevated free cholesterol in cells from macrophage-specific ABCA1 knockout mice enhances the pro-inflammatory response of macrophages, suggesting that ABCA1 may attenuate the inflammatory response at the cellular level [26]. In addition, SREBP2 was found to positively regulate ABCA1 promoter activity and promote ABCA1 expression [27]. Therefore, we speculated that SREBP2 might alter cell motility by regulating ABCA1 in asthma. In contrast to the above reports, we found that TGF-β1 decreased ABCA1 levels in ASMCs, and ABCA1 is a target gene of SREBP2. Specifically, overexpression of ABCA1 alleviated the SREBP2-induced increase or decrease in intracellular parameters, but it was unable to change the expression of SREBP2 in AMSCs.

The Toll-like receptor family is a class of protein molecules closely related to the inflammatory response [28]. NF-κB is involved in a variety of signaling pathways during inflammation [28]. Inhibition of TLR4/NF-κB signaling pathway activation reduces microglial inflammation [29]. NFATc1 is an important member of the nuclear factor family of activated T cells and primarily affects lymphocyte proliferation and TH2-type immune responses in airway inflammation [30,31]. The imbalance of Th1/Th2 immune response plays an important role in the development of asthma [30]. Loss of NFATc1 results in impaired lymphocyte proliferation and inhibits lymphocyte differentiation [32,33]. Therefore, we speculated that TLR2/NF-κB/NFATc1 pathway may be involved in SREBP2 regulation in asthma. In this study, we found that SREBP2 could activate TLR2/NF-κB/NFATc1, and ABCA1 could inhibit SREBP2-induced activation, illustrating that SREBP2 may increase inflammation and airway remodeling. Yuan et al. reported that activation of NF-κB signaling pathway leads to increased lung inflammation, alveolar damage, and airway remodeling, which is consistent with the findings of this study and further confirms that SREBP2 has a proinflammatory effect in asthma [34].

## Conclusion

This study verified that SREBP2 activates TLR2/NF-κB/NFATc1 regulatory network and promotes TGF-β1-induced cell motility by inhibiting ABCA1 expression. Although research has found that miR-30b-5p promotes ASMC dysfunction by activating the PI3K/AKT pathway [16], our study provides different regulators against ASMCs, providing potential therapeutic targets and new perspectives for the treatment of asthma. However, *in vivo* animal experiments are needed in future studies to deeply validate the role of SREBP2 and ABCA1 in asthma.

## Data Availability

All data generated or analyzed during this study are included in this published article.

## Highlight

- TGF-β1 increased SREBP2 level and decreased ABCA1 level
- SREBP2 knockdown inhibited proliferation, migration and promoted apoptosis
- SREBP2 activated the TLR2/NF-κB/NFATc1 pathway through inhibiting ABCA1 expression
- SREBP2 promoted TGF-β1-induced cell motility through regulating ABCA1 expression

## References

[1] Poon AH, Hamid Q. Severe asthma: have we made progress?. Ann Am Thorac Soc. 2016;13(Suppl 1):S68. 27027956 10.1513/AnnalsATS.201508-514MG

[2] Han F, Li S, Yang Y, et al. Interleukin-6 promotes ferroptosis in bronchial epithelial cells by inducing reactive oxygen species-dependent lipid peroxidation and disrupting iron homeostasis. Bioengineered. 2021;12:5279–5288. 34402724 10.1080/21655979.2021.1964158 PMC8806540

[3] Krishnan JA, Lemanske RF, Canino GJ, et al. Asthma outcomes: symptoms. J Allergy Clin Immunol. 2012;129:S124–S135. 22386505 10.1016/j.jaci.2011.12.981 PMC4263029

[4] Nunes C, Pereira AM, Morais-Almeida M. Asthma costs and social impact. Asthma Res Pract. 2017;3(1):1. 28078100 10.1186/s40733-016-0029-3 PMC5219738

[5] James AL, Elliot JG, Jones RL, et al. Airway smooth muscle hypertrophy and hyperplasia in asthma. Am J Respir Crit Care Med. 2012;185:1058–1064. 22403800 10.1164/rccm.201110-1849OC

[6] Akinbami LJ, Moorman JE, Bailey C, et al. Trends in asthma prevalence, health care use, and mortality in the United States, 2001-2010. NCHS Data Brief. 2012;94:1–8. 22617340

[7] Ma J, Wang Q, Fei T, et al. MCP-1 mediates TGF-beta-induced angiogenesis by stimulating vascular smooth muscle cell migration. Blood. 2007;109:987–994. 17032917 10.1182/blood-2006-07-036400

[8] Horton JD, Goldstein JL, Brown MS. SREBPs: activators of the complete program of cholesterol and fatty acid synthesis in the liver. J Clin Invest. 2002;109:1125–1131. 11994399 10.1172/JCI15593 PMC150968

[9] Jeon TI, Osborne TF, Tem M. SREBPs: metabolic integrators in physiology and metabolism. Trends Endocrinol Metab. 2012;23:65–72. 22154484 10.1016/j.tem.2011.10.004 PMC3273665

[10] Guo C, Chi Z, Jiang D, et al. Cholesterol homeostatic regulator SCAP-SREBP2 Integrates NLRP3 inflammasome activation and cholesterol biosynthetic signaling in macrophages. Immunity. 2018;49:842–856.e7. 30366764 10.1016/j.immuni.2018.08.021

[11] Lee W, Ahn JH, Park HH, et al. COVID-19-activated SREBP2 disturbs cholesterol biosynthesis and leads to cytokine storm. Signal Transduct Target Ther. 2020;5. DOI:10.1038/s41392-020-00292-7 PMC7471497 32883951

[12] Almstrand AC, Josefson M, Bredberg A, et al. TOF-SIMS analysis of exhaled particles from patients with asthma and healthy controls. Eur Respir J. 2012;39(1):59–66. 21719486 10.1183/09031936.00195610

[13] Soumian S, Albrecht C, Davies AH, et al. ABCA1 and atherosclerosis. Vasc Med. 2005;10:109–119. 16013195 10.1191/1358863x05vm593ra

[14] Bodzioch M, Orsó E, Klucken J, et al. The gene encoding ATP-binding cassette transporter 1 is mutated in Tangier disease. Nat Genet. 1999;22:347–351. 10431237 10.1038/11914

[15] Zeng L, Liao H, Liu Y, et al. Sterol-responsive element-binding protein (SREBP) 2 down-regulates ATP-binding cassette transporter A1 in vascular endothelial cells: a novel role of SREBP in regulating cholesterol metabolism. J Biol Chem. 2004;279:48801–48807. 15358760 10.1074/jbc.M407817200

[16] Wang W, Guo J, Wang Y. MicroRNA-30b-5p promotes the proliferation and migration of human airway smooth muscle cells induced by platelet-derived growth factor by targeting phosphatase and tensin homolog deleted on chromosome ten. Bioengineered. 2021;12:3662–3673. 34251961 10.1080/21655979.2021.1950401 PMC8806833

[17] Livak KJ, Schmittgen TD. Analysis of relative gene expression data using real-time quantitative PCR and the 2(-Delta Delta C(T)) Method. Methods. 2001;25:402–408. 11846609 10.1006/meth.2001.1262

[18] Yan F, Wufuer D, Ding J, et al. MicroRNA miR-146a-5p inhibits the inflammatory response and injury of airway epithelial cells via targeting TNF receptor-associated factor 6. Bioengineered. 2021;12:1916–1926. 34002665 10.1080/21655979.2021.1927545 PMC8806598

[19] Yang ZH, Bi-Wen MO. Role of Airway Smooth Muscle Cells in Asthma Mechanisms. World Latest Med Inf. 2018.

[20] Royce SG, Cheng V, Samuel CS, et al., The regulation of fibrosis in airway remodeling in asthma. Mol Cell Endocrinol. 2012;351:167–175. 22266540 10.1016/j.mce.2012.01.007

[21] Chen M, Huang L, Zhang W, et al. MiR-23b controls TGF-β1 induced airway smooth muscle cell proliferation via TGFβR2/p-Smad3 signals. Mol Immunol. 2016;70:84–93. 26748386 10.1016/j.molimm.2015.12.012

[22] ouwaop Maknd. The regulatory role of TGF- in airway remodeling in asthma. Immunol Cell Biol. 2007;85(5): 348–356. 17325694 10.1038/sj.icb.7100044

[23] Qiang Y, Chen Y, Han L, et al. Inflammatory stress increases unmodified LDL uptake via LDL receptor: an alternative pathway for macrophage foam-cell formation. Inflammation Res. 2009;58:809. 10.1007/s00011-009-0052-4 19533020

[24] Li LC, Varghese Z, Moorhead JF, et al. Cross-talk between TLR4-MyD88-NF-κB and SCAP-SREBP2 pathways mediates macrophage foam cell formation. Am J Physiol Heart Circ Physiol. 2013;304:H874–884. 23335792 10.1152/ajpheart.00096.2012

[25] Oram JF, Vaughan AM. ATP-binding cassette cholesterol transporters and cardiovascular disease. Circ Res. 2006;99:1031–1043. 17095732 10.1161/01.RES.0000250171.54048.5c

[26] Zhu X, Lee JY, Timmins JM, et al. Increased cellular free cholesterol in macrophage-specific Abca1 knock-out mice enhances pro-inflammatory response of macrophages. J Biol Chem. 2008;283:22930–22941. 18552351 10.1074/jbc.M801408200 PMC2516976

[27] Jenny W, Quinn CM, Brown AJ. SREBP-2 positively regulates transcription of the cholesterol efflux gene, ABCA1, by generating oxysterol ligands for LXR. Biochem J. 2006;400(3): 485–491. 16901265 10.1042/BJ20060914 PMC1698594

[28] Boyd J, Mathur H, Sumeet W, et al. Toll-like receptor stimulation in cardiomyoctes decreases contractility and initiates an NF-κB dependent inflammatory response. Cardiovasc Res. 2006;72:384–393. 17054926 10.1016/j.cardiores.2006.09.011

[29] Zhang X-Y, Liu Y, He T, et al. Anaphylatoxin C5a induces inflammation and reduces insulin sensitivity by activating TLR4/NF-kB/PI3K signaling pathway in 3T3-L1 adipocytes. Biomed Pharmacothe. 2018;103:955–964. 10.1016/j.biopha.2018.04.057 29710512

[30] Desai D, Brightling C. Cytokines and cytokine-specific therapy in asthma. Adv Clin Chem. 2012;57:57–97. 22870587 10.1016/b978-0-12-394384-2.00003-6

[31] Peng SL, Gerth AJ, Ranger AM, et al. NFATc1 and NFATc2 together control both T and B cell activation and differentiation. Immunity. 2001;14:13–20. 11163226 10.1016/s1074-7613(01)00085-1

[32] Oh-Hora M, Rao A. Calcium signaling in lymphocytes. Curr Opin Immunol. 2008;20:250–258. 18515054 10.1016/j.coi.2008.04.004 PMC2574011

[33] Said SI, Hamidi SA, Gonzalez Bosc L. Asthma and pulmonary arterial hypertension: do they share a key mechanism of pathogenesis? Eur Respiratory J Off J Eur Soc Clin Respiratory Physiol. 2010;35:730. 10.1183/09031936.00097109 PMC2963099 20356986

[34] Yuan FAB, Liu RC, Hu MA, et al. JAX2, an ethanol extract of Hyssopus cuspidatus Boriss, can prevent bronchial asthma by inhibiting MAPK/NF-κB inflammatory signaling. Phytomedicine. 2019;57:305–314. 30807985 10.1016/j.phymed.2018.12.043

